# High-Dose Ambroxol Therapy in Type 1 Gaucher Disease Focusing on Patients with Poor Response to Enzyme Replacement Therapy or Substrate Reduction Therapy

**DOI:** 10.3390/ijms24076732

**Published:** 2023-04-04

**Authors:** Majdolen Istaiti, Dafna Frydman, Tama Dinur, Jeff Szer, Shoshana Revel-Vilk, Ari Zimran

**Affiliations:** 1 Gaucher Unit, Shaare Zedek Medical Center, Jerusalem 9103102, Israel; dafnaf@szmc.org.il (D.F.); dinurtama@gmail.com (T.D.); svilk@szmc.org.il (S.R.-V.); azimran@gmail.com (A.Z.); 2 Peter MacCallum Center, Royal Melbourne Hospital, Department of Medicine, University of Melbourne, Melbourne, VIC 3050, Australia; jeff.szer@mh.org.au; 3 Faculty of Medicine, Hebrew University, Jerusalem 91120, Israel

**Keywords:** Gaucher disease (GD), chaperone therapy, enzyme replacement therapy (ERT), substrate reduction therapy (SRT), ambroxol, suboptimal response, Lyso-Gb1

## Abstract

Ambroxol hydrochloride (ABX), an oral mucolytic drug available over the counter for many years, acts as a pharmacological chaperone for mutant glucocerebrosidase, albeit at higher doses. Proof-of-concept reports have been published over the past decade on all three types of Gaucher disease (GD). Here, we assess the safety and efficacy of 12 months of 600 mg ambroxol per day in three groups of Type 1 GD patients with a suboptimal response to enzyme replacement therapy (ERT) or substrate reduction therapy (SRT), defined as platelet count < 100 × 10^3^/L, lumbar spine bone density T-score < −2.0, and/or LysoGb1 > 200 ng/mL, and for a group of naïve patients who had abnormal values in two of these three parameters. We enrolled 40 patients: 28 ERT- or SRT-treated, and 12 naïve. There were no severe adverse effects (AEs). There were 24 dropouts, mostly due to AEs (*n* = 12), all transient, and COVID-19 (*n* = 7). Among the 16 completers, 5 (31.2%) had a >20% increase in platelet count, 6 (37.5%) had a >0.2 increase in T-score, and 3 (18.7%) had a >20% decrease in Lyso-Gb1. This study expands the number of patients exposed to high-dose ABX, showing good safety and satisfactory efficacy, and provides an additional rationale for adding off-label ABX to the arsenal of therapies that could be offered to patients with GD1 and a suboptimal response or those unable to receive ERT or SRT.

## 1. Introduction

Type 1 Gaucher disease (GD1) is one of the two most common lysosomal storage disorders. Safe and effective therapy has been available for GD1 in the form of intravenous enzyme replacement therapy (ERT) since 1991 for all patients, and in the form of oral substrate reduction therapy (SRT) since 2000 for only adult patients [1,2,3]. Most patients respond favorably, with improvement in blood counts (particularly platelets and hemoglobin), a reduction in hepatosplenomegaly, improvement in bone manifestations (symptoms and imaging findings), and overall improved quality of life [4]. The success of these specific therapies was assessed in different ways, including the achievement of GD specific therapeutic goals [5,6]; more recently, we and colleagues recommended to consider normalization as a more ambitious treatment goal [6,7].

The use of biomarkers has added an important response measure to therapies, as by definition, they are more sensitive than traditional clinical measures are [8]. Among the different biomarkers, the most specific and sensitive biomarker is glucosyl sphingosine (Lyso-Gb1), which has been used in our routine follow-up of all patients with GD since 2014 [9,10].

Despite the impressive impact of both ERT and SRT on the clinical course of GD1, some patients fail to achieve the therapeutic goals or normalization [7]. The general propensity to reporting trials with positive results may account for the paucity of publications addressing the issue of poor response. One example of such a report is a publication linking focal splenic lesions to refractory thrombocytopenia [11]. However, a careful reading of almost all the reports of interventional clinical trials indicates cases in whom there has been inadequate or total lack of improvement; among the many different causes are neutralizing antibodies in the case of ERT [12] and advanced disease manifestations (such as massive fibrosis of liver or spleen or irreversible skeletal complications).

The steps that treating physicians can take when facing poor responders include increased doses in cases of patients receiving lower ERT doses, switching from one ERT to another (particularly switching from a mutant to a wild-type ERT [4,13]), changing modality (ERT to SRT or SRT to ERT [14]), or possibly combining modalities (ERT and SRT [15]).

With the recognition that GD is not just a glycolipid storage disease, but also a misfolding protein disorder [16], a new therapeutic modality of pharmacological chaperones (PC) has emerged [17]. Following the failure of the first trial of isophagomine [18], Maegawa and Mahoren identified ambroxol hydrochloride (ABX) as a suitable PC and thereby a potential new treatment modality for patients with the most GD genotypes [19], although at a higher dose than that used as an antitussive. Since then, several studies and case reports have documented the safety and efficacy of high-dose ABX in patients with GD1, neuronopathic GD (nGD), and even with GBA-related Parkinson’s disease (PD) [20,21,22].

In this study, we report on a prospective open-label clinical trial (investigator-initiated research (IIR)) on the use of high-dose ABX as an adjuvant to ERT or SRT in patients with GD1 who had suboptimal or no response to therapy. During the course of the study, the protocol was amended to include another arm of ABX as a single agent.

## 2. Results

### 2.1. Patients

A total of 40 patients (21 males) at the median age of 54 (24–84) years, 22 heterozygous for N370S mutations (55%), were recruited for the study (Table 1 and Table S1). A total of 26 patients on ERT received a median monthly dose of 49 (25–150) units/kg for a median period of 19 (3–32) years; 2 patients received SRT: one received 100 mg miglustat three times per day for a period of 3.5 years, and the second received eliglustat at a dose of 84 mg twice daily for a period of 2 years; 12 naïve patients entered the study and received at least a dose of ABX.

**Figure 1:**
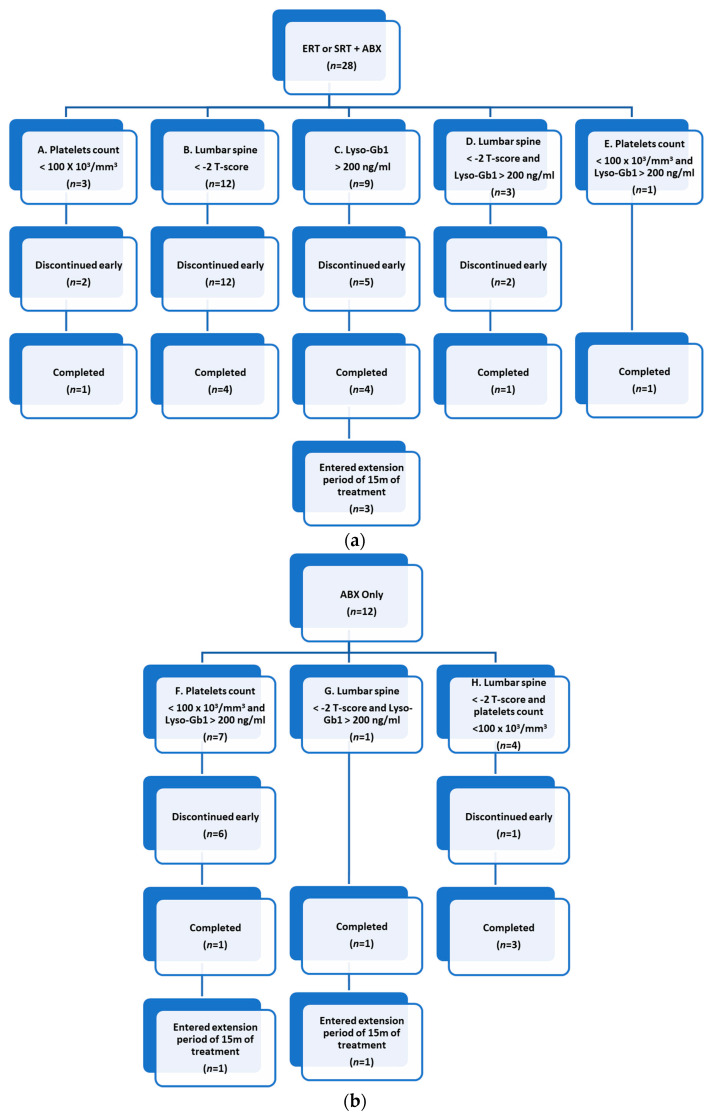
Enrolment groups, dropouts, and study completion of (**a**) patients with suboptimal/poor response to ERT or SRT and (**b**) patients naïve to ERT or SRT, i.e., never received (*n* = 2) or have been off therapy for at least 12 months (*n* = 10). The group categories are outlined from A to H.

Of the 40 patients, 16 completed the study; all demonstrated near 100% compliance, as measured by counting the empty blister packs. In total, 24 patients discontinued ABX therapy prior to study completion: withdrawn due to adverse events (AEs) (*n* = 12), COVID-19 (*n* = 7), the inability to swallow ABX capsules (*n* = 3), a new diagnosis of PD and advised to receive a higher dose of ABX [22] (*n* = 1), and participation in another clinical trial (*n* = 1). Patient disposition is presented in Figure 1.

### 2.2. Safety

Of the 40 enrolled patients, 13 reported AEs (Table 2). None of the AEs was severe: one patient had moderate AE (cough), and the other AEs were mild; all AEs were transient. Of the 15 AEs, 3 were reported by the patient prior to enrollment in the study, but were defined as AEs due to the investigator’s assessment of the worsening intensity or frequency of the complaints. Among the routine laboratory parameters evaluated for all patients at each hospital visit, there was one case of a transient asymptomatic elevation of the liver enzymes that rapidly resolved upon the discontinuation of ABX. Eight patients developed hypouricemia (uric acid < 2.6 mg/dL).

### 2.3. Efficacy

A total of 16 patients completed the study. Of the 6 patients who had entered the study from the thrombocytopenia group, 3 achieved the primary outcome of greater than 20% improvement in platelet counts (mean, 41.5%; two from the ERT or SRT group, and one from the naïve group). In addition, two patients from the ERT or SRT group (one from the Lyso-Gb1 group and one from the dual-energy X-ray absorptiometry (DEXA) group) who had 157 × 10^9^/L and 127 × 10^9^/L, respectively, demonstrated greater than 20% increase in platelet counts: 24.2% and 31.5%, respectively. Overall, 5 out of the 16 completers (31.2%) achieved greater than 20% improvement in platelet counts (Table S2).

Of the 11 patients who had entered the study from the DEXA group, 5 achieved the primary outcome of greater than 0.2 improvement in lumbar spine T-score (two on ERT or SRT and three naïve). In addition, another patient with minimal osteopenia (T-score −1.1), improved by 0.4 in 12 months (moving from the osteopenia category to normal bone density) Overall, 6 out of the 16 completers achieved great than 0.2 improvement in lumbar spine T-score (Table S3).

Eight patients entered the study from the Lyso-Gb1 group (6 from the ERT or SRT group and 2 from the naïve group). Of those, only 2 patients achieved a greater than 20% reduction in Lyso-Gb1 levels, 53.5% and 35% each. In addition, one patient from the naïve group achieved a 30% reduction in Lyso-Gb1 from the baseline of 132 ng/mL. Overall, 3 out of the 16 completers achieved greater than 20% reduction in Lyso-Gb1 (Table S4). Interestingly, there were 4 patients with a significant reduction in Lyso-Gb1 levels at Month 1 with a dosage of 150 mg/day, 3 of which with a baseline Lyso-Gb1 greater than 200 ng/mL. However, the Lyso-Gb1 levels increased with time so the last value at the end of the study did not meet the desired outcome (Table S4).

**Figure 2:**
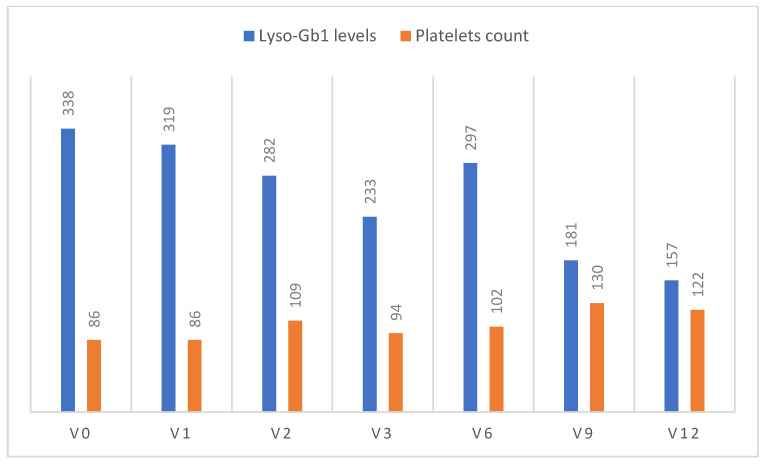
Changes in platelet counts and Lyso-Gb1 levels from visit (V) 0 (enrollment) to the end of the study (V12) for Case 1.

In order to emphasize the efficacy of ABX, we present two patients with good response, one from each group (ERT or SRT and naïve):

Case 1—E1 (Figure 2): A 24-year-old Ashkenazi Jewish patient was diagnosed with severe GD1 in early childhood after presenting with massive splenomegaly, anemia, and thrombocytopenia. The diagnosis was confirmed via the low enzyme activity and molecular diagnosis of RecTL/N307S. The family history included a father with early-onset and rapidly progressive PD (age 40). ERT with imiglucerase was started in 1998, and due to the poor platelet response and no improvement in bone density, her dose was increased to 120 U/kg/month, with no improvement. After 12 years, she was switched to velaglucerase alfa, which she received for 8 years, and then switched to eliglustat 2 tablets daily, again showing no change in platelet count (86 × 10^9^/L), Lyso-Gb1 levels (338 ng/mL) and T-score (−2.2). With this background, she joined this IIR. Figure 2 shows the increase in platelet count and decrease in Lyso-Gb1 levels. DEXA was improved to a T-score of −1.9. At 12 months, she was advised to continue with commercial ABX, but there was a temporary hiatus until she was able to access the ABX product; during these 3 months, there was a drop in platelet count and an increase in Lyso-Gb1. Both parameters improved again with the reinstitution of ABX treatment.

**Figure 3:**
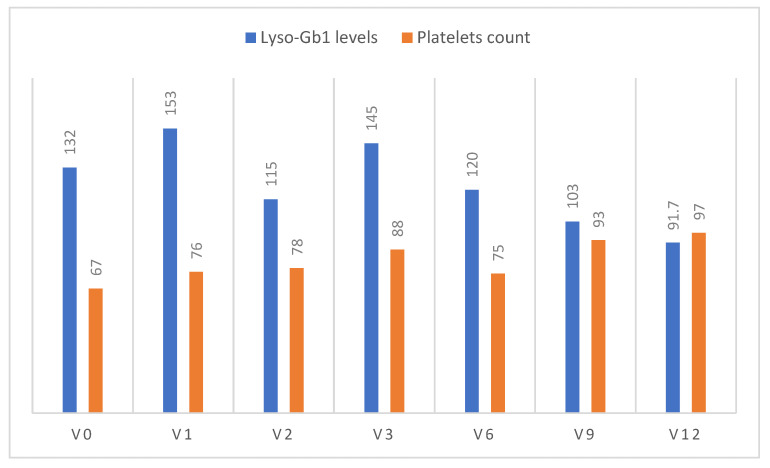
Changes in platelet count and Lyso-Gb1 levels from visit (V) 0 (enrollment) to the end of the study (V12) for Case 2.

Case 2—H4 (Figure 3): A 55-year-old mother of 12 children was diagnosed with symptomatic GD1 in 1989. Clinical features were massive splenomegaly and severe thrombocytopenia, with a history of postpartum hemorrhage in almost every pregnancy. Molecular analysis revealed the common Ashkenazi Jewish genotype of N370S/N370S. For many years, she refused treatment due to her inability to cope with the biweekly infusions. In 2017, she presented with severe abdominal pain, possibly related to splenic infarcts, and she agreed to start ERT, receiving velaglucerase alfa infusions for 8 months. Unfortunately, after each infusion, she suffered severe back pain, and was thereby switched to eliglustat despite the improvement in spleen volume and platelet count. This also resulted in significant AEs, so she remained untreated for 3 years. She joined the naïve arm of this study and experienced no AEs. Figure 3 shows the improvement in platelet count, and the decrease in Lyso-Gb1 levels. The DEXA T-score improved from −2.3 to −2.0. She decided to continue treatment with commercial ABX. Interestingly, this patient also had a family history of early-onset PD.

## 3. Discussion

This IIR is the first attempt to use ABX as an adjuvant therapy to ERT or SRT in a cohort of patients with GD1 who showed poor or no response to these specific modalities. In fact, even among the naïve patients, 10 of the 12 had received ERT or SRT, but either developed significant AEs (as in Case 2) or failed to show a satisfactory response; therefore, they remained untreated. The two other naïve patients were unwilling to receive ERT or SRT. With this background, what seems to be the partial success of ABX (~40% success for the platelets and DEXA, and 20% for the Lys-Gb1) is actually quite impressive and justifies further study. Adding to the efficacy data is the exceptional safety profile, as we did not observe any previously unknown AEs [20], and there were no serious or severe drug-related AEs. The laboratory AE of the reduction in the levels of uric acid detected in 8 out of all patients (20%), although asymptomatic, might be of some concern due to the fact that hypouricemia was reported in association with PD [23]. On the other hand, ABX may play a beneficial role in the management of GD-related PD and maybe even for the prevention of PD [24].

Unfortunately, ABX is a common generic drug manufactured by many different pharmaceutical companies, none of which is currently motivated to invest in clinical trials for GD (not even for GBA1-related PD [20]. In these circumstances, it is up to treating physicians to consider adding ABX or switching to ABX alone in patients with suboptimal or no response to ERT or SRT, respectively. One could also consider using ABX as a single-agent PC for patients who are unable or unwilling to receive ERT or SRT, or maybe even in patients with mild phenotypes who do not fulfill the criteria used in their countries for specific therapy. ABX has the additional advantage of cost-effectiveness if healthcare providers are willing to reimburse for this medication. While significantly cheaper than ERT or SRT, giving the required high dose for GD places a financial burden on individual patients that may be challenging to meet.

ABX is currently studied (also IIRs) as a PC therapy for patients with GBA1-related and idiopathic PD [22], and in GBA1-related dementia [25]; it will soon be studied for the prevention of GBA1-related PD in individuals with advanced prodromal features [26,27]. Given the high prevalence of PD among patients with GD, the administration of oral ABX might have added value. Both cases presented above had a family history of PD, as we find in approximately 25% of our patients with GD [28].

There are several limitations to this study, with the most significant one being the relatively few completers. This was directly and indirectly related to COVID-19. While 7 patients (17.5%) dropped out directly due to the pandemic, additional patients may have elected to withdraw from the study due to an AE that could have normally been dealt with differently. Additional limitations are related to the dosage and the mode of administration, none of which has been traditionally studied during drug development. The selection of the dose was somewhat arbitrary, and it may be that 600 mg/day is either too high or not high enough. One approach, described in Fabry disease, could be to use smaller doses than 600 mg/day or alternate daily dosing [29]. However, other than the few patients who showed reductions in Lyso-Gb1 after 1 month (see Table S4), we do not have evidence from this study to support lower doses nor have we seen supportive data for such a change in the majority of the patients of our pilot study in 2011 [30]. A higher dose than 600 mg/day (as commonly prescribed in nGD and PD), is probably unnecessary in GD1, as there is no need to cross the blood–brain barrier (BBB). To reach 600 mg, patients had to take 8 capsules daily, another limiting factor and cause for dropout. During the course of the study, one Israeli pharmacy compounded 200 and 300 mg capsules of ABX; however, these were only used temporarily by a single patient (Case 1). Another limitation is the absence of in vitro testing as the prescreening of patients, as was reported by other investigators [21,22]. We do not have clear evidence from the literature that demonstrates a relationship between the change in enzymatic activity in the fibroblasts following exposure to ABX in vitro and the magnitude of response in the patients. In fact, there are contradicting results in homozygous patients for L444P [31]. Since we were dealing a priori with patients who had failed to adequately respond to ERT or SRT, adding ABX, with its known excellent safety profile, seemed to be justified.

## 4. Materials and Methods

The study was conducted at the Gaucher unit, SZMC from 17 March 2019 to 9 November 2022, enrolling adult patients with GD1 (>18 years): 28 with a suboptimal/poor response to ERT or SRT, and 12 naïve to treatment, i.e., never treated or off treatment for at least 12 months prior to enrollment. GD diagnosis was confirmed in all patients via low enzymatic activity, a biallelic mutation based on the whole *GBA1* sequence, and elevated Lyso-Gb1 levels [32]. For the 28 suboptimal responders, ABX was added to the current ERT or SRT.

A suboptimal response to ERT or SRT with no change in the dose of ERT or SRT in the previous 12 months was defined as one or more of the following: platelet count < 100 × 10^9^/L, DEXA lumbar spine T-score < −2.0, or Lyso-Gb1 > 200 ng/mL. Treatment-naïve patients were required to have had at least two of the above abnormalities to be eligible. Exclusion criteria included comorbidities that may have impacted the primary and/or secondary outcomes, pregnant or lactating women, the inability to cooperate with the study procedures (unwillingness or inability to swallow 8 capsules a day), hypersensitivity to ABX, and participation in another clinical trial.

The primary endpoints were an increase of 20% in platelet counts, an increase of 0.2 in DEXA T-score at 12 or 15 months, or a decrease of 20% or more in Lyso-Gb1 levels. Safety was assessed by capturing AEs.

All routine laboratory tests were conducted locally at SZMC other than for Lyso-Gb1, which was assayed at Centogene, Rostock, Germany, as previously described [33]. The DEXA was conducted locally using our standard Hologic DXA unit (Hologic, Marlborough, MA, USA). Ambroxol hydrochloride was provided as 75 mg retarded-slow-release capsules (Heumann Pharma, Nurnberg, Germany). For the first month, the daily dose was 150 mg (comparable with the usual dosage for cough suppression); in the second month, the dose was doubled to 300 mg daily; starting from the third month, the full daily dose of 600 mg was administered until the end of study. In five patients who had failed to achieve any benefit at 6 months, ABX 600 mg was administered alternate-daily for a period of another 6 months starting at Month 9.

The initial institutional review board (IRB) approval was received on 9 July 2018 and for three other amendments: the addition of naïve patients; the addition of SRT, as the original design addressed patients on ERT only; the addition of a 9-month visit and the extension of the study to 15 months in patients who had failed to achieve satisfactory results at 6 months, and in whom the administration of ABX had been changed from daily to alternate-daily. The protocol was registered as ClinicalTrials.gov identifier NCT03950050. Written informed consent was obtained from all participants prior to initiating the study-related procedures and for each of the three amendments.

### Statistical Analysis

To summarize the descriptive statistics, we used the median (range) for the continuous variables. For the nominal data, we report the absolute and relative frequencies.

## 5. Conclusions

Our study expanded the number of patients exposed to high-dose ABX, showing good safety and efficacy. It provides an additional rationale for adding off-label ABX to the arsenal of therapies that could be offered to patients with GD1 with a suboptimal response or those unable to receive ERT or SRT. Further research is needed to define the optimal ABX regimens with regard to both the dose and frequency of administration.

## Figures and Tables

**Table 1:** Clinical characteristics of patients included in the study.

	Total	Completers
Number	40	16
Sex, male (%)	21 (53)	8 (50)
Age (years) *	54 (24–84)	52 (24–84)
*GBA1* variants ** (%)		
- N370S/N370S	18 (45)	9 (56)
- N370S/other	22 (55)	7 (44)
Naïve patients (%)	12 (30)	5 (31)
- GD specific therapies		
- Single ERT	8	3
- ERT X 2	13	7
- ERT X 3	4	-
- ERT X 2 + SRT	2	1
- ERT X 3 + SRT	1	-
Years on GD specific therapies *	20.5 (3–32)	18 (3–28)

* Median (range). ** Old nomenclature. GD, Gaucher disease; ERT, enzyme replacement therapy; SRT, substrate reduction therapy.

**Table 2:** Adverse events (AEs) associated with ABX.

Adverse Event	Number of Patients *	Patient’s Group **
Abdominal pain/diarrhea	2	A and D
Nausea	1	B
Cough	1	C
Depression/nightmares	3	B, C and F
Atypical chest pain	1	B
Vertigo/headaches/dizziness	3	A, D and F
Elevated liver enzymes	1	B
Hypertension	1	F
Elevated Lyso-Gb1	2	D and F

* Two patients reported two different AEs: one had elevated Lyso-Gb1 and vertigo, and another reported headaches and anxiety; all the others had a single AE. ** Patient group categories (adjuvant or naïve) were derived from Figure 1.

## Data Availability

Data cannot be shared due to ethical and privacy issues.

## Supplementary Materials

The supporting information can be downloaded at: https://www.mdpi.com/article/10.3390/ijms24076732/s1.
