# Supplementary material for: High-Dose Ambroxol Therapy in Type 1 Gaucher Disease Focusing on Patients with Poor Response to Enzyme Replacement Therapy or Substrate Reduction Therapy
Source: Int J Mol Sci. 2023 Apr 4;24(7):6732. doi: 10.3390/ijms24076732 (PMC10095311; doi:10.3390/ijms24076732)
Supplement: Supplementary file 1 [file ijms-24-06732-s001.zip › ijms-2312214-supplementary.pdf]

Table S1. Characteristics of all patients.

GROUP/ NUMBER	GENDER	AGE	GENOTYPE	PREVIOUS GD SPECIFIC TREATMENTS	LAST DOSE OF ERT/SRT	LENGTH OF GD SPECIFIC THERAPIES (YEARS)
A1	M	68	N370S/N370S	OGT918 – imi- **vela**	120 u/kg/month	20
A2	M	39	N370S/IVS2+1	imi- vela – eli– **vela**	150 u/kg/month	32
A3*	M	38	N370S/RecTERT	imi– **vela**	60 u/kg/month	12
B1	F	59	N370S/N370S	imi- OGT918 - vela- eli– **mig**	100mgX3/day	26
B2	F	71	N370S/N370S	**vela**	28 u/kg/month	5
B3*	F	72	N370S/N370S	imi– **vela**	34 u/kg/month	16
B4*	F	84	N370S/N370S	**vela**	30 u/kg/month	4
B5	M	60	N370S/N370S	**vela**	30 u/kg/month	6
B6*	M	43	N370S/N370S	imi– **vela**	66 u/kg/month	12
B7	F	52	N370S/L444P	imi– **vela**	25 u/kg/month	26
B8	F	70	N370S/V394L	imi– **vela**	34 u/kg/month	21
B9	F	69	N370S/IVS2+1	**vela**	37 u/kg/month	9
B10*	F	56	N370S/L444P	**Vela**	60 u/kg/month	3
B11	M	60	N370S/L444P	tali – **vela**	130 u/kg/month	9
B12	M	57	N370S/L444P	imi – tali – **vela**	50 u/kg/month	24
C1	F	54	N370S/84GG	imi– **vela**	60 u/kg/month	26
C2*	F	54	N370S/L444P	imi– **vela**	30 u/kg/month	28
C3	M	69	N370S/L444P	imi– **vela**	28 u/kg/month	11
C4	M	52	N370S/84GG	imi– **vela**	48 u/kg/month	28
C5	M	45	N370S/55bpdeletion	imi - tali – **vela**	60 u/kg/month	26
C6*	M	40	N370S/del55	imi– **vela**	56 u/kg/month	24
C7	M	67	N370S/N370S	imi – tali - **vela**	30 u/kg/month	17
C8*	M	57	N370S/N370S	Imi– **vela**	60 u/kg/month	18
C9*	M	69	N370S/N370S	imi– **vela**	30 u/kg/month	28
D1	F	59	N370S/84GG	**Imi**	70 u/kg/month	26
D2*	M	24	N370S/IVS2+1	**Vela**	106 u/kg/month	22
D3	F	39	N370S/RecTL	**Imi**	60 u/kg/month	15
E1*	F	24	N370S/RecTL	imi -vela – **eli**	84mgX2/day	22
F1	F	37	N370S/N370S	None	NA	NA
F2	F	45	N370S/N370S	None	NA	NA
F3*	F	64	N370S/N370S	None	NA	NA
F4	M	38	N370S/N370S	None	NA	NA
F5	F	61	N370S/IVS2+1	None	NA	NA
F6	M	43	N370S/84GG	None	NA	NA
F7	M	29	N370S/84GG	None	NA	NA
G1*	M	46	N370S/V394L	None	NA	NA
H1	M	39	N370S/N370S	None	NA	NA
H2*	M	25	N370S/N370S	None	NA	NA

H3*	F	50	N370S/N370S	None	NA	NA
H4*	F	55	N370S/N370S	None	NA	NA

Abbreviations: imi, imiglucerase; vela, velaglucerase alfa; tali, taliglucerase alfa; eli, eliglustat; mig, miglustat.

*Completers

Table S2. Platelet count for all 16 patients who completed the study.

Number	***Baseline***	***Month 1***	***Month 2***	***Month 3***	***Month 6***	***Month 9***	***Month 12***	***Month 15***
A3	**95**	**101**	**82**	**102**	**114**	**132**	**131**	
E1	**86**	**86**	**109**	**94**	**102**	**130**	**122**	
C8	360	373	328	369	374	391	362	353
C9	316	292	301	353	307	328	279	324
C6	**157**	**191**	**168**	**172**	**191**	**184**	**195**	**182**
C2	349	285	334	297	347	326	373	
D2	299	305	270	288	310	293	269	
B3	128	124	123	124	140	145	123	
B4	272	225	247	255	271		275	
B6	**127**	**151**	**136**	**149**	**129**		**167**	
B10	235	230	251	246	252	238	246	
F3	41	34	38	33	29	32	32	33
H3	41	33	29	32	35		41	
H4	**67**	**76**	**78**	**88**	**75**	**93**	**97**	
H2	97	85	97	86	112		89	
G1	104	93	110	113	120	118	119	113

Table S3. BMD T score for all 16 patients who completed the study.

Number	*Baseline*	*Month 12*	*Month 15*
B3	**-2.3**	**-2.1**	
B4	-2.7	-3.2	
B6	-2.6	-2.7	
B10	-2.3	-2.5	
D2	-3.3	-3.7	
E1	**-2.2**	**-1.9**	
C8	-1.7		-1.6
C9			
C6	**-1.1**	**-0.7**	
C2	-1.7	-2.2	
A3	-1.8	-1.7	

G1	**-2.5**		**-2.2**
H3	-2.4	-2.6	
H4	-2.3	-2	
H2	-2.2	-2.5	
F3	-3		-2.7

Table S4. Lyso-Gb1 levels for all 16 patients who completed the study.

Number	*Baseline*	*Month 1*	*Month 2*	*Month 3*	*Month 6*	*Month 9*	*Month 12*	*Month 15*
E1	**338**	**319**	**282**	**233**	**297**	**181**	**157**	
C8	249	189	204	194	236	240	225	231
C9	225	141	197	251	319	272	205	288
C6	251	277	318	265	336	183	235	277
C2	**559**	**613**	**507**	**599**	**447**	**407**	**363**	
D2	787	716	761	617	687	699	698	
B3	22.8	20.2	23.4	22.3	20.2	16.8	26.6	
B4	65.3	73.4	73.1	50.3			40.1	
B6	142	133	115	121	85.6		110	
B10	12.5	10.1	11	10.2	16.5	15.1	9.6	
A3	54	52.8	52.2	63.7	62	66.3	69.7	73.7
F3	205		229	222	239	242	258	376
G1	331	281	228.9	325	309	411	327	426
H3	175	193	182	242	237		222	
H4	132	153	115	145	120	103	91.7	
H2	155	120	144	180	140		177	
